# Canadians’ use of cannabis for therapeutic purposes since legalization of recreational cannabis: a cross-sectional analysis by medical authorization status

**DOI:** 10.1186/s12916-024-03370-7

**Published:** 2024-04-08

**Authors:** Lynda G. Balneaves, Ashleigh Brown, Matthew Green, Erin Prosk, Lucile Rapin, Max Monahan-Ellison, Eva McMillan, Jonathan Zaid, Michael Dworkind, Cody Z. Watling

**Affiliations:** 1 College of Nursing, Rady Faculty of Health Sciences, University of Manitoba, Winnipeg, MB Canada; 2 SheCann Cannabis, Winnipeg, MB Canada; 3 Medical Cannabis Canada, Toronto, ON Canada; 4 Department of Psychology, Brock University, St. Catharine’s, ON Canada; 5 Santé Cannabis, Montréal, QC Canada; 6 Department of Medicine, McGill University, Montréal, QC Canada

**Keywords:** Medical cannabis, Policy, Symptoms, Decision support, Experience, Recreational cannabis, Authorization, Program

## Abstract

**Background:**

There has been a precipitous decline in authorizations for medical cannabis since non-medical cannabis was legalized in Canada in 2018. This study examines the demographic and health- and medical cannabis-related factors associated with authorization as well as the differences in medical cannabis use, side effects, and sources of medical cannabis and information by authorization status.

**Methods:**

Individuals who were taking cannabis for therapeutic purposes completed an online survey in early 2022. Multivariable logistic regression was used to determine odds ratios (OR) and 95% confidence intervals (CI) of demographic and health- and medical cannabis-related variables associated with holding medical cannabis authorization. The differences in medical cannabis use, side effects, and sources of information by authorization status were determined via *t*-tests and chi-squared analysis.

**Results:**

A total of 5433 individuals who were currently taking cannabis for therapeutic purposes completed the study, of which 2941 (54.1%) currently held medical authorization. Individuals with authorization were more likely to be older (OR ≥ 70 years vs. < 30 years, 4.85 (95% CI, 3.49–6.76)), identify as a man (OR man vs. woman, 1.53 (1.34–1.74)), have a higher income (OR > $100,000/year vs. < $50,000 year, 1.55 (1.30–1.84)), and less likely to live in a small town (OR small town/rural vs. large city, 0.69 (0.59–0.81)). They were significantly more likely to report not experiencing any side effects (29.9% vs. 23.4%; *p* < 0.001), knowing the amount of cannabis they were taking (32.1% vs. 17.7%; *p* < 0.001), obtaining cannabis from regulated sources (74.1% vs. 47.5%; *p* < 0.001), and seeking information about medical cannabis from healthcare professionals (67.8% vs. 48.2%; *p* < 0.01) than individuals without authorization.

**Conclusions:**

These findings offer insight into the possible issues regarding equitable access to medical cannabis and how authorization may support and influence individuals in a jurisdiction where recreational cannabis is legalized, highlighting the value of a formal medical cannabis authorization process.

**Supplementary Information:**

The online version contains supplementary material available at 10.1186/s12916-024-03370-7.

## Background

Taking cannabis for therapeutic purposes, hereafter referred to as medical cannabis, has been a growing phenomenon in Canada, with an increasing body of evidence indicating that it may help with numerous symptoms and health conditions including, but not limited to, pain, spasticity, chemotherapy-induced nausea and vomiting, epilepsy, and sleep disorders [1–3]. Since 2001, Canadians could obtain cannabis for medical purposes through the Medical Marihuana Access Regulations, which permitted individuals to receive authorization from a physician or a nurse practitioner to possess dried cannabis for certain indications when conventional therapies were deemed unsuccessful or inappropriate. Since that time, several regulations have come into effect, including the Access to Cannabis for Medical Purposes Regulations (ACMPR) in 2016 that permitted the legal sale of dried cannabis, cannabis oils, and fresh cannabis materials and allowed individuals to personally produce or designate someone to grow their own cannabis. In 2018, the Canadian government legalized the non-medical (or “recreational”) use of cannabis for adults with the passage of the Cannabis Act and Regulations [4]. The ACMPR was subsumed under the Cannabis Act, with no substantial changes made to medical access.

The Canadian Cannabis Survey (CCS) estimates that 13% of Canadians aged 16 years or older, or over one million individuals in Canada, take cannabis to manage symptoms associated with a disease or health condition [5]. As of June 2023, however, there were only 203,933 active medical authorizations through a registered licensed seller of medical cannabis [6], a decline of 40% since October 2018. Understanding why Canadians are not obtaining authorization, particularly since the legalization of recreational cannabis, is urgently needed to identify the personal, social, and structural factors that may be influencing the decrease in access to medical cannabis through the formal authorization process. It is also important to understand the potential implications of accessing medical cannabis through a non-healthcare route in which there may be limited to no consultation and follow-up with a healthcare professional.

The aim of this study was threefold: (1) identify the demographic factors associated with holding medical cannabis authorization; (2) identify the associations between the reasons and the health conditions for which Canadians use medical cannabis, as well as their history of medical cannabis use, and holding medical cannabis authorization; and (3) examine the differences between Canadians holding authorization versus those who do not with regard to their use of medical cannabis and sources of medical cannabis information.

## Methods

### Study design/setting

We conducted a cross-sectional survey of Canadians who were currently taking medical cannabis through the legal medical cannabis program and/or other sources (i.e., legal and illegal recreational sources). Eligible participants were 16 years or older, able to read English or French, and were Canadian residents. Recruitment occurred through social media (i.e., Facebook, Instagram, Twitter), newsletters, website postings, and emails from partner organizations (e.g., Arthritis Society Canada, Santé Cannabis, Medical Cannabis Canada, SheCann Cannabis). Taking cannabis for medical purposes was defined at the beginning of the survey as “any use of cannabis to treat or manage a symptom, side effect, or health condition.”

The survey was available online via Qualtrics© from March 1, 2022, to July 31, 2022. Enhanced security detection was implemented to ensure responses were from valid participants. Survey completion took a median of 28 min, and participants could enter a random draw to win 1 of 20 $50 gift cards.

### Primary measurements/outcomes

The questionnaire was modified from a prior survey conducted in 2020 by Medical Cannabis Canada (https://patientaccess.ca/survey/) following consultation with study partners and was piloted with 10 individuals with medical cannabis experience. Beta testing of the survey was undertaken with ~ 200 members of SheCann Cannabis, a national patient advocacy group. The final survey comprised 90 items. Questions included demographic information including age, province or territory of residence, ethnicity, size of city or town they live in, gender, education obtained, household income, and if they were a member of the Canadian Armed Forces. Further details regarding the classification and description of these items can be found in the Additional file 1: Supplementary Methods. For this study, the following variables/items were examined:

- *Medical cannabis use and authorization*—Participants were asked to select from a variety of descriptions that best described their use of medical cannabis. Those who selected “I am currently taking cannabis for medical purposes with a document from a doctor or nurse practitioners authorizing its use” or “I am currently taking cannabis for medical purposes without a document from a doctor or nurse practitioner authorizing its use” were categorized as current medical cannabis consumers. Participants who indicated they currently had a document authorizing medical cannabis use were considered to have current authorization to take medical cannabis.
- *When medical cannabis authorization obtained*—Participants who indicated they currently held medical cannabis authorization were asked when they had received a medical document from a healthcare provider to access medical cannabis. Response options included < 6 months, 6 months to < 1 year, 1 to < 3 years, 3 to < 5 years, 5 to < 10 years, and 10 years or over ago.
- *Health issue medical cannabis taken for*—Participants were asked what symptoms or health conditions they managed with medical cannabis and could select all that apply from a list of 27 health issues. An open-ended option was also available for participants to specify a symptom or health condition not listed.
- *Duration of medical cannabis use*—Participants were asked “How long have you been taking medical cannabis?” Options included < 6 months, 6 months to < 1 year, 1 to < 2 years, 2 to < 3 years, 3 to < 5 years, 5 to < 10 years, and 10 years or over. Participants were categorized into each respective group.
- *Reasons for taking medical cannabis*—Participants were asked “What are some of the reasons why you take medical cannabis?” and could select from a list of 14 reasons and/or specify in an open textbox a reason, if not listed. These reasons included medical cannabis working well in managing their health issue; enhancing the effect of, or working better, than other medication/treatment; giving them a sense of control over their health; having fewer side effects; being a natural treatment; being readily available through recreational cannabis stores; or suggested by someone they trust. A full list of reasons asked about in the questionnaire can be found in Additional file 1: Supplementary Methods.
- *Type of medical cannabis product*—Participants were asked to indicate which types of medical cannabis they take for each health condition they reported being managed by medical cannabis. Options included dried flower, oils, capsules or soft gels, oral sprays, edibles, topicals, concentrates, vape, beverages, suppositories, or oral strips.
- *Amount of medical cannabis taken*—Participants were asked to estimate how much tetrahydrocannabinol (THC) and/or cannabidiol (CBD) they take on average per day for each type of medical cannabis product they utilized. As the unit of measurement can vary across types of product (e.g., dried flower, oil, spray), participants were able to estimate using milligrams, milliliters, or percentages. Participants could also indicate “I don’t know” if they could not estimate the amount of THC or CBD consumed.
- *Medical cannabis side effects*—Participants were asked “What, if any, unwanted side effects have you experienced from taking medical cannabis?” They could select all that applied from a list of 14 common side effects (e.g., dry mouth, feeling tired, unable to concentrate) or specify a side effect if not listed. A full list of side effects asked about in the questionnaire can be found in Additional file 1: Supplementary Methods.
- *Source(s) of medical cannabis*—Participants were asked where they purchased/obtained medical cannabis products and could select all that applied from a list of nine regulated and unregulated sources across the medical and recreational cannabis markets.
- *Source(s) of medical cannabis information*—Participants were asked “In the past 12 months, from which of the following individuals have you sought out or received information about medical cannabis?” Options included a variety of healthcare professionals as well as “friend, family, or acquaintance”; “dealer”; or “none of the above.” In addition, participants were asked to select other locations that they have sought or received information about medical cannabis, including walk-in clinics, medical cannabis clinics (in-person and online), recreational cannabis stores, support groups, websites (hospital, government/university, patient advocacy), search engines (e.g., Google, Yahoo, Bing), research journals, media, social media, and books/magazines. An open-ended option allowed participants to write in a source not listed.

### Statistical analysis

Descriptive statistics summarized participants’ demographic information, separating individuals with current authorization versus those without.

For study objectives 1 and 2, multivariable-adjusted logistic regression analyses, with authorization status as the outcome, were conducted to assess which variables were associated with holding current authorization. Demographic variables including age, gender, ethnicity, size of city and town of residence, obtained education, yearly household income, and being a current or former member of the Canadian Armed Forces were added to multivariable models, adjusting for province or territory of residence and each of the other variables. We also explored via multivariable-adjusted logistic regression models the health issues medical cannabis was taken for, duration of medical cannabis use, and reasons for taking medical cannabis that were associated with holding current authorization, after adjusting for the above demographic factors. Participants with unknown or missing information for a specific variable were categorized into a missing/unknown category for the logistic regression analyses. For specific health issues medical cannabis was taken for, univariable logistic regression models with authorization as the outcome were explored first and only symptoms and/or health conditions that were significantly associated with holding current medical authorization were added in multivariable-adjusted logistic regression models.

For study objective 3, simple comparisons between those with authorization and those without authorization were conducted using χ^2^ (for categorical variables) and *t*-tests (for normally distributed continuous variables). Specifically, we examined the differences in those with authorization versus those without on the types of cannabis products they took, side effects experienced, and sources of medical cannabis and information utilized. Due to a relatively low percentage of missing data (< 5%), participants who did not answer a specific question of interest were excluded from the respective analysis. Bonferroni correction was applied to descriptive statistics tests to limit the risk of type I error, and *p*-values < 0.001 (0.05/49 comparisons) were considered statistically significant.

### Ethics, consent, and permissions

Ethical approval was obtained from the University of Manitoba’s Research Ethics Board (HE2021-0156 and HE2022-0149) and McGill Faculty of Medicine and Health Sciences Research Ethics Board (21-12-025). All participants provided electronic consent before beginning the online survey.

## Results

A total of 5433 participants reported currently taking medical cannabis. Nearly 62% identified as a woman, with most respondents reporting their ethnicity as White (81.3%), with 5.7% identifying as Indigenous, and 5.8% reporting mixed ethnicity. Participants ranged in age from 16 to 89 years (mean = 49.5 years (SD = 14.4 years)). Almost 93% reported achieving a high school education or higher, whereas close to 30% reported a before-tax household income of < $35,000 CAD/year (Table 1).

**Table 1:** Demographics of current medical cannabis consumers—with and without authorization

	**Currently taking medical cannabis**		**All**
	**With authorization**	**Without authorization**	
**Number of participants**	2941 (54.1)	2492 (45.9)	5433
**Age, mean (SD)**	52.3 (13.6)	46.3 (14.4)	49.5 (14.4)
**Gender, *N* (%)**			
Male	1127 (39.0%)	646 (26.3%)	1773 (33.1%)
Female	1665 (57.6%)	1638 (66.6%)	3303 (61.7%)
Non-binary	70 (2.4%)	142 (5.8%)	212 (4.0%)
**Province/territory, *N* (%)**			
British Columbia	295 (10.2%)	436 (17.7%)	731 (13.7%)
Alberta	464 (16.0%)	313 (12.7%)	777 (14.5%)
Saskatchewan	72 (2.5%)	95 (3.9%)	167 (3.1%)
Manitoba	132 (4.6%)	147 (6.0%)	279 (5.2%)
Ontario	1020 (35.3%)	792 (32.2%)	1812 (33.9%)
Québec	551 (19.1%)	209 (8.5%)	760 (14.2%)
New Brunswick	105 (3.6%)	119 (4.8%)	224 (4.2%)
Nova Scotia	186 (6.4%)	225 (9.1%)	411 (7.7%)
Prince Edward Island	17 (0.6%)	32 (1.3%)	49 (0.9%)
Newfoundland	40 (1.4%)	72 (2.9%)	112 (2.1%)
Yukon	3 (0.1%)	7 (0.3%)	10 (0.2%)
Northwest Territories	3 (0.1%)	9 (0.4%)	12 (0.2%)
Nunavut	4 (0.1%)	4 (0.2%)	8 (0.1%)
**Ethnicity, *N* (%)**			
Arab	8 (0.3%)	3 (0.1%)	11 (0.2%)
Asian	14 (0.5%)	9 (0.4%)	23 (0.4%)
Black	32 (1.1%)	13 (0.5%)	45 (0.8%)
Indigenous	98 (3.4%)	215 (8.7%)	313 (5.9%)
Latin American	16 (0.6%)	15 (0.6%)	31 (0.6%)
South Asian (e.g., Indian, Pakistani)	15 (0.5%)	9 (0.4%)	24 (0.4%)
West Asian (e.g., Iranian)	2 (0.1%)	3 (0.1%)	5 (0.1%)
White	2404 (83.2%)	1940 (78.9%)	4344 (81.2%)
Not listed	83 (2.9%)	55 (2.2%)	138 (2.6%)
Mixed	150 (5.2%)	160 (6.5%)	310 (5.8%)
Prefer not to say	67 (2.3%)	37 (1.5%)	104 (1.9%)
**Education, *N* (%)**			
No diploma or degree	108 (3.7%)	169 (6.9%)	277 (5.2%)
High school	540 (18.7%)	657 (26.7%)	1197 (22.4%)
Trade certificate or diploma	282 (9.8%)	232 (9.4%)	514 (9.6%)
College	911 (31.5%)	760 (30.9%)	1671 (31.2%)
University certificate	248 (8.6%)	159 (6.5%)	407 (7.6%)
Undergraduate degree	510 (17.6%)	307 (12.5%)	817 (15.3%)
Graduate degree	241 (8.3%)	125 (5.1%)	366 (6.8%)
**Income, *N* (%)**			
< $35,000	700 (24.2%)	791 (32.2%)	1491 (27.9%)
$35,000–$50,000	437 (15.1%)	470 (19.1%)	907 (17.0%)
$50,001–$75,000	490 (16.9%)	393 (16.0%)	883 (16.5%)
$75,001–$100,000	410 (14.2%)	272 (11.1%)	682 (12.7%)
$100,001–$150,000	389 (13.5%)	237 (9.6%)	626 (11.7%)
> $150,000	192 (6.6%)	107 (4.4%)	299 (5.6%)

Values are *N* (%) unless otherwise indicated. Values include missing information and therefore may not add up to 100% due to these missing values

*N* Number of participants, *SD* Standard deviation

Of the total sample, 54.1% reported holding current medical cannabis authorization. Close to three-quarters of individuals with current authorization (73.8%) reported first obtaining authorization more than 3 years ago. The most prevalent health conditions for which medical cannabis was taken were chronic pain (67.0%), anxiety (63.6%), and sleep issues (61.8%) (Additional file 1: Table S1). The average number of health indications for which medical cannabis was taken was five (SD = 3.4).

### Demographic and health-related factors associated with holding medical cannabis authorization

Participants who were older were more likely to report holding authorization (≥ 70 years vs. < 30 years: odds ratio (OR), 4.85 (95% confidence intervals (CI), 3.49–6.76), *p*-trend < 0.001), as were participants who identified as a man (man vs. woman: OR, 1.53 (1.34–1.74)). Further, those who reported having undergraduate (OR, 1.62 (1.33–1.98)) or graduate education (OR, 1.56 (1.19–2.04)) in comparison with high school education were more likely to report holding medical cannabis authorization, as were individuals who received a household income higher than $75,000 CAD (OR, 1.40 (1.16–1.70)) and $100,000 CAD (OR, 1.55 (1.30–1.84)) in comparison with those whose household income was less than $50,000 CAD (Table 2). In addition, participants who identified as being members of the Canadian Armed Forces, including Veterans, were three times as likely to report being authorized to take medical cannabis versus no military affiliation (OR 3.06 (2.19–4.27)). With regard to ethnicity, individuals who identified as Indigenous were half as likely to report holding authorization (OR, 0.49 (0.38–0.64) in comparison with individuals who identified as White. Individuals living in small, rural towns were also less likely to report holding medical cannabis authorization (OR, 0.69 (0.59–0.81)) than those living in large cities.

**Table 2:** Multivariable adjusted odds ratios (95% CI) of demographic characteristics associated with current authorization status

**Demographic factors**	**Odds ratio (95% confidence interval)**
**Age**	
< 30 years	1 (ref)
30–39.9 years	**2.03 (1.57–2.63)**
40–49.9 years	**2.39 (1.85–3.10)**
50–59.9 years	**2.71 (2.09–3.52)**
60–69.9 years	**4.01 (3.08–5.21)**
≥ 70 years	**4.85 (3.49–6.76)**
**Gender**	
Woman	1 (ref)
Man	**1.53 (1.34–1.74)**
Non-binary	0.76 (0.55–1.06)
**Education**	
No diploma or degree	0.75 (0.56–1.00)
High school	1 (ref)
College, trade certificate, or diploma	1.24 (1.07–1.45)
Undergraduate degree	**1.62 (1.33–1.98)**
Graduate degree	**1.56 (1.19–2.04)**
**Household income**	
< $50,000 per year	1 (ref)
$50,001–75,000 per year	1.24 (1.05–1.47)
$75,001–100,000 per year	**1.40 (1.16–1.70)**
$100,001+ per year	**1.55 (1.30–1.84)**
**Member of the Canadian Armed Forces**—Yes	**3.06 (2.19–4.27)**
**Ethnicity**	
White	1 (ref)
Black	1.68 (0.85–3.34)
Asian	1.20 (0.65–2.21)
Indigenous	**0.49 (0.38–0.64)**
Latin American	0.89 (0.41–1.91)
Mixed	1.04 (0.81–1.33)
**City/town**	
Large city	1 (ref)
Medium city	0.78 (0.64–0.95)
Small city	0.80 (0.68–0.94)
Small town/rural	**0.69 (0.59–0.81)**

All variables are adjusted for each other as well as the province/territory of where the participant resided. Values in bold represent statistically significant findings where *p* < 0.001 with a value > 1.0 indicating being more likely to hold authorization whereas a value < 1.0 indicating being less likely to hold authorization

*CI* Confidence intervals, *ref* Reference group

In terms of health- and medical cannabis-related factors, individuals who reported taking medical cannabis to manage depression were less likely to report holding current authorization (OR, 0.78 (0.67–0.90)) whereas participants who reported taking medical cannabis to address chronic pain (OR, 1.74 (1.50–2.01)), seizures (OR, 1.88 (1.19–2.98)), and traumatic brain injury (TBI) (OR, 2.12; CI, 1.50–3.02) were more likely to report holding authorization (Table 3). In addition, participants who reported taking medical cannabis for 3–10 years were more likely to report holding authorization (OR, 1.56 (1.07–2.27)) than those who were within the first 6 months of taking medical cannabis (Table 3). However, participants who had the lengthiest experience with medical cannabis (over 10 years) were found to be half as likely to report holding current authorization compared to the most recent consumers of medical cannabis (< 6 months; OR, 0.54, 0.37–0.79) (Table 3).

**Table 3:** Multivariable adjusted odds ratios (95% CI) of health- and medical cannabis-related factors associated with current authorization status

**Health- and medical cannabis-related factors**	**Odds ratio (95% confidence interval)**
**Health issues medical cannabis taken for**^**a**^	
ADHD	0.84 (0.69–1.01)
Agitation	0.88 (0.72–1.06)
Anxiety	0.83 (0.71–0.98)
Appetite	0.96 (0.81–1.13)
Bipolar disorder	1.05 (0.78–1.42)
Concentration	1.02 (0.83–1.24)
** Depression**	**0.78 (0.67–0.90)**
Pain—acute	0.91 (0.77–1.08)
** Pain—chronic**	**1.74 (1.50–2.01)**
Migraine	1.20 (1.03–1.39)
Muscle spasms	1.08 (0.93–1.25)
Nausea and vomiting	0.80 (0.68–0.95)
PCOS	0.90 (0.62–1.30)
PTSD	1.25 (1.06–1.47)
** Seizures**	**1.88 (1.19–2.98)**
Stress	1.09 (0.94–1.26)
** Traumatic brain injury**	**2.12 (1.50–3.02)**
**Duration of medical cannabis use**	
< 6 months	1 (ref)
6 months to < 1 year	0.74 (0.46–1.19)
1 to < 2 years	1.19 (0.78–1.81)
2 to < 3 years	0.86 (0.58–1.28)
3 to < 5 years	**1.56 (1.07–2.27)**
5 to < 10 years	**1.54 (1.06–2.26)**
10+ years	**0.54 (0.37–0.79)**
**Reasons for taking medical cannabis**^**b**^	
In addition to other medications.	1.09 (0.95–1.24)
**To reduce the use of other medications.**	**1.30 (1.14–1.48)**
**To reduce side effects of other medications/treatments.**	**1.29 (1.11–1.51)**
**Other medications do not work well for me**.	**1.28 (1.11–1.48)**
General health and wellness.	0.97 (0.84–1.11)
**For recreational purposes**.	**0.49 (0.42–0.56)**
**Works well managing health condition(s) or symptom(s).**	**1.62 (1.37–1.91)**
**Enhances effect of other medication(s).**	**1.40 (1.18–1.66)**
**It works better than other medications or treatments I take.**	**1.55 (1.35–1.77)**
It is a natural treatment.	1.06 (0.93–1.22)
**Gives control over my health.**	**1.34 (1.18–1.53)**
**Less expensive than other medications I take.**	**0.51 (0.43–0.61)**
Fewer side effects than other medication(s) I take.	1.14 (0.99–1.31)
**I can purchase it at a recreational store.**	**0.28 (0.24–0.32)**

Multivariable adjusted odds ratios are adjusted for ethnicity, gender, age, province/territory currently live, size of city or town, education, annual household income, being a member of the Canadian Armed Forces, and health conditions or symptoms cannabis was taken for

Values in bold represent statistically significant findings where *p* < 0.001 with a value > 1.0 indicating being more likely to hold authorization whereas a value < 1.0 indicating being less likely to hold authorization

*CI* Confidence intervals, *ref* Reference group

^a^The reference group for each consists of individuals without the condition/symptom

^b^The reference group consists of individuals who indicated they did not use medical cannabis for a specific reason. Reasons were not mutually adjusted for each other in multivariable models

Numerous reasons for taking medical cannabis were found to be associated with holding current authorization, such as perceiving cannabis to be effective, including more so than other medications, and potentiating the effect of existing medications (Table 3). In contrast, participants were found to be less likely to hold current authorization if they reported taking cannabis, at times, for recreational purposes, perceived medical cannabis to be less expensive than other medications, and the fact they could purchase cannabis for therapeutic purposes from a recreational store (Table 3).

Individuals with authorization were significantly more likely (*p* < 0.001) to report taking oils and capsules while those without authorization were more likely to report using dried flower, edibles, and concentrates (*p* < 0.001) (Table 4). Participants generally struggled to estimate the amount of medical cannabis they utilized; however, participants with authorization were more likely to be able to report the amount of cannabis they took daily, including THC and CBD content, compared to individuals without authorization (32.1% vs. 17.7%; *p* < 0.001) (data not shown).

**Table 4:** Differences in medical cannabis use and side effects experienced by authorization status

	**Currently taking medical cannabis**		***p*-value for difference**
	**With authorization**	**Without authorization**	
**Types of products participants reported taking**^**a**^			
**Dried flowe**r	**1917 (66.3%)**	**1938 (78.8%)**	**< 0.001**
**Oils**	**1978 (68.4%)**	**1086 (44.2%)**	**< 0.001**
**Edibles**	**1388 (48.0%)**	1531 (62.3%)	**< 0.001**
Vape	1156 (40.0%)	1052 (42.8%)	0.036
**Capsules**	**1006 (34.8%)**	**521 (21.2%)**	**< 0.001**
**Topical**	**802 (27.7%)**	**588 (23.9%)**	**< 0.001**
**Concentrates**	**630 (21.8%)**	**637 (25.9%)**	**< 0.001**
**Oral spray**s	**506 (17.5%)**	**276 (11.2%)**	**< 0.001**
**Beverages**	**352 (12.2%)**	**373 (15.2%)**	**< 0.001**
**Oral strips**	**171 (5.9%)**	**46 (1.9%)**	**< 0.001**
**Suppositories**	**130 (4.5%)**	**61 (2.5%)**	**< 0.001**
**Reported side effects associated with current medical cannabis consumption**^**b**^			
Anxiety	251 (8.7%)	230 (9.4%)	0.006
Confusion	126 (4.4%)	119 (4.9%)	0.027
**Cough**	**684 (23.7%)**	**861 (35.1%)**	**< 0.001**
**Dependency or addiction to cannabis**	**105 (3.6%)**	**264 (10.8%)**	**< 0.001**
**Dry mouth**	**1235 (42.8%)**	**1168 (47.6%)**	**< 0.001**
Feeling faint	113 (3.9%)	89 (3.6%)	0.58
Feeling intoxicated	401 (13.9%)	361 (14.7%)	0.012
**Feeling paranoid**	**170 (5.9%)**	**208 (8.5%)**	**< 0.001**
Feeling tired	563 (19.5%)	544 (22.2%)	0.058
Nausea	68 (2.4%)	62 (2.5%)	0.81
Rapid heart rate	201 (7.0%)	213 (8.7%)	0.066
Trouble remembering things	434 (15.0%)	443 (18.1%)	0.011
Unable to concentrate	216 (7.5%)	189 (7.7%)	0.037
**Vomiting**	**12 (0.4%)**	**31 (1.3%)**	**< 0.001**
**I have not experienced any side effects**	**861 (29.9%)**	**573 (23.4%)**	**< 0.001**

^a^Values are the number of participants who reported taking the medical cannabis product

^b^Values are *N* (%), representing the number of participants who reported experiencing the specific side effect

Participants with authorization were more likely to report that they had not experienced any side effects associated with cannabis use compared to those without authorization (29.9% vs. 23.4%; *p* < 0.001). The latter were more likely to report experiencing cough (35.1% vs. 23.7%; *p* < 0.001), dependency/addiction (10.8% vs. 3.6%; *p* < 0.001), dry mouth (47.6% vs. 42.8%; *p* < 0.001), and feeling paranoid (8.5% vs. 5.9%; *p* < 0.001) (Table 4).

### Differences in sources of medical cannabis and information by authorization status

**Fig. 1:**
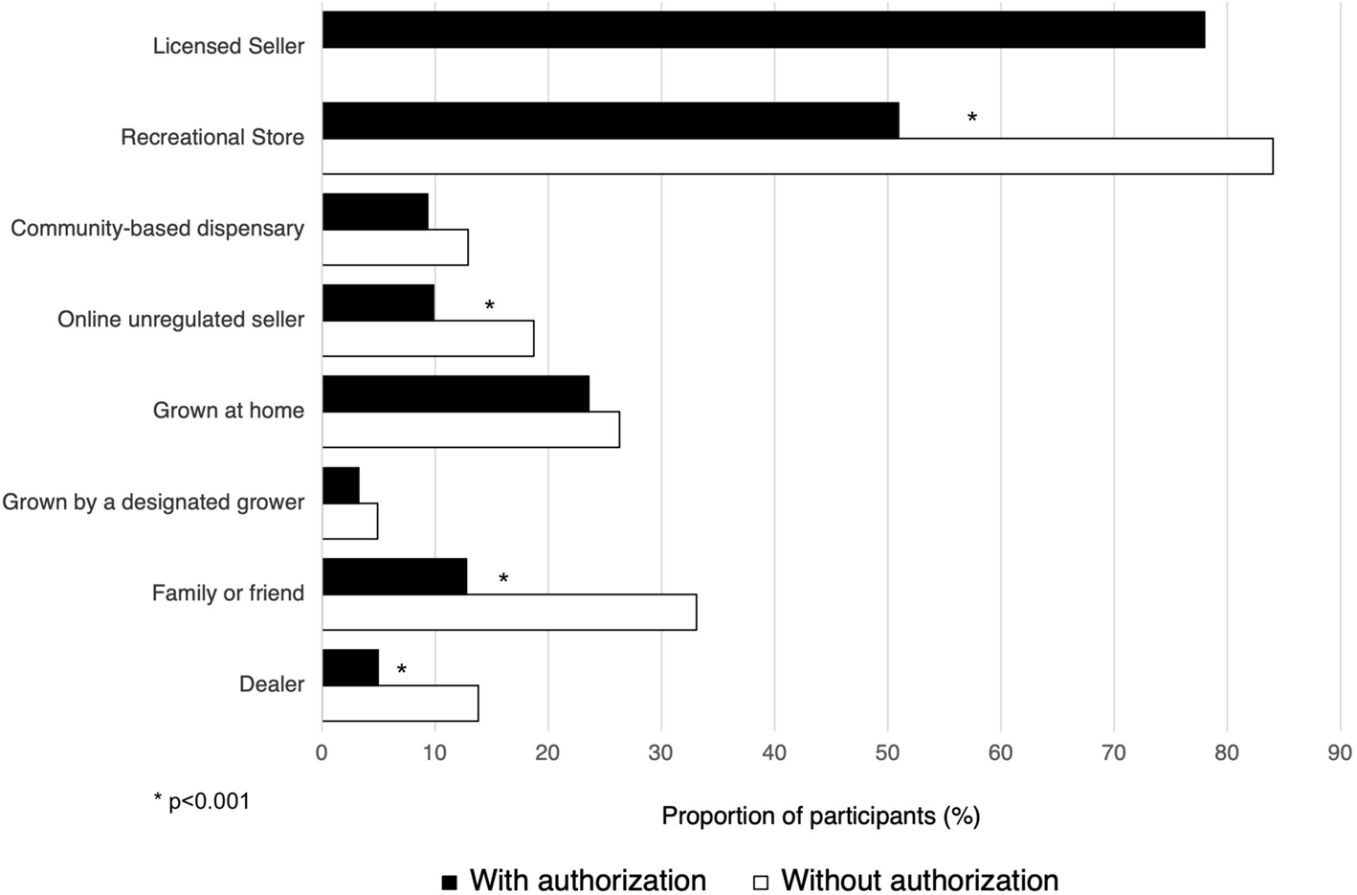
Sources of medical cannabis in the past 12 months separated by authorization status. *p < 0.001

With regard to the source of medical cannabis, a total of 3395 individuals (62.5%) reported obtaining cannabis from the legal recreational market. Those with authorization were significantly more likely to get cannabis from only legal or regulated sources (including medical and recreational) than those without authorization (74.1% vs. 47.5%; *p* < 0.001); however, only 1040 individuals (35.4%) with authorization reported obtaining cannabis exclusively from their authorized sources. Individuals without authorization were significantly more likely (*p* < 0.001) to access the non-legal market or other unregulated sources, such as family and friends, dealers, and online unregulated sellers than those without authorization (Fig. 1).

**Fig. 2:**
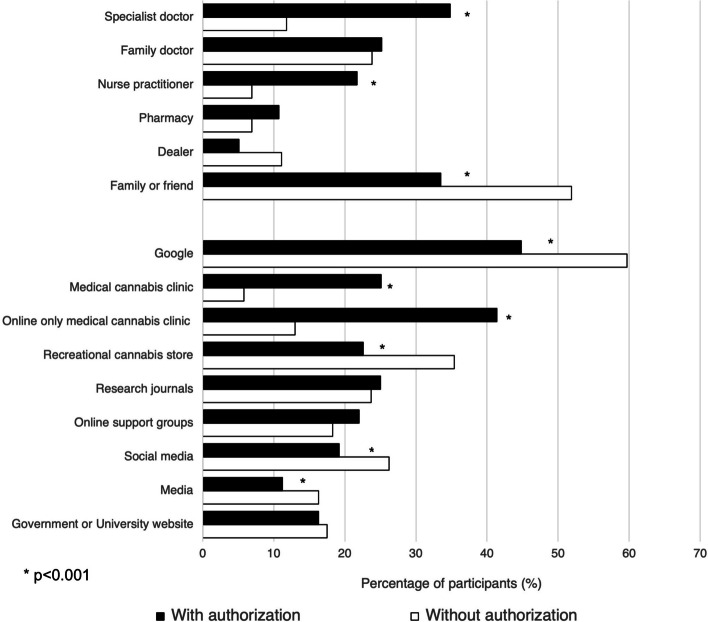
Sources of information on medical cannabis separated by medical authorization status. *p < 0.001

Participants with authorization were significantly more likely to get information from the following sources: a specialist doctor, a nurse practitioner, a medical cannabis clinic, or an online support group than those without authorization (*p* < 0.001). In contrast, participants without authorization were more likely to receive information from family or friends, a dealer, Google, recreational cannabis stores, or the media compared to those with authorization (*p* < 0.001) (Fig. 2).

## Discussion

Numerous demographic and health-related factors were associated with holding medical cannabis authorization, including older age, identifying as a man, higher household income, having a university education, living in a large city, and taking medical cannabis for chronic pain, seizures, or traumatic brain injury. Several differences were also observed between individuals who held authorization versus those who did not have authorization, including their source and types of medical cannabis products, their awareness regarding how much cannabis they consumed, the symptoms and health conditions they took medical cannabis for, the side effects they experienced, and their information sources.

To our knowledge, there have been no studies conducted in Canada that have examined the demographic and health-related factors associated with medical cannabis authorization and the differences in medical cannabis utilization by authorization status since the legalization of non-medical cannabis. The limited research that does exist [7–10] has found similar associations identified in our study between individuals with authorization and those without, including age, ethnicity, education attainment, income, and medical cannabis access sources. These associations point to potential equity issues regarding access to informed healthcare professionals who are willing to discuss and provide authorization for medical cannabis, as well as the affordability of cannabis through licensed sellers (i.e., courier costs, taxes), and public awareness of the medical cannabis program in Canada [11]. For example, Veterans being three times as likely to hold authorization may be a result of the reimbursement policy and available information about medical cannabis provided by Veterans Affairs [12].

Our findings suggest that medical authorization, which requires a consultation with a healthcare professional, may lead to better-informed medical cannabis consumers who are less likely to experience side effects, are more certain about the amount of medical cannabis they are consuming, and are more likely to access cannabis through regulated sources. While access through the recreational market may be perceived by both individuals and healthcare professionals as easier in terms of time and bureaucracy [13, 14], it may come with a cost in terms of lack of access to evidence-based information about medical cannabis, including appropriate product and dosing guidance. Individuals who bypass the medical cannabis access program may also not be informed about the potential health risks and benefits of medical cannabis in the context of their personal health history or be exposed to misinformation from unqualified individuals [14–16]. The fact that half of the individuals with authorization in our study were accessing medical cannabis through recreational sources raises concerns that reasonable access to cannabis via the medical program, a constitutionally protected right [17], has not been achieved. With the legalization of recreational cannabis in Canada, healthcare professionals may be more inclined to suggest individuals access medical cannabis through the legal recreational market. For example, in the 2022 Health Canada survey of 823 healthcare professionals, 70% recommended individuals access medical cannabis through a recreational store [13]. Healthcare professionals may also lack sufficient education, training, and knowledge to feel comfortable authorizing medical cannabis use for patients [18].

Our study findings offer timely direction regarding how the medical cannabis landscape in Canada could be re-envisioned to promote equity and reasonable access, as well as to support informed treatment decisions by Canadians. Pharmacies as a point of access for medical cannabis have been discussed for many years in Canada and other jurisdictions [19, 20] and may facilitate individuals to receive evidence-based advice, particularly regarding contraindications and drug-cannabis interactions [21]. Lastly, ongoing evaluation of whether reasonable access to medical cannabis is being achieved, and by whom, as well as the impact of the legalization of the recreational market on medical cannabis access, is urgently needed to inform future policy decisions and priorities. Future research exploring and understanding the barriers and inequities surrounding medical cannabis use and access, and the related outcomes, is imperative as cannabis policies in Canada continue to evolve.

## Limitations

The study findings must be viewed with caution due to several limitations. As an observational, cross-sectional study, the associations observed between study variables may be due to factors that were not measured or accounted for in the survey. This may be particularly important as individuals who had medical authorization in our study had a higher education than individuals without authorization, which may contribute to some of the observed associations. Participants were recruited through convenience sampling; thus, the sample may not be representative of the larger medical cannabis community in Canada. A selection bias may exist in which individuals who were willing and able to participate and complete an online survey may be different than individuals who decline such an opportunity. The results presented were not weighted on factors such as region, sex, or age based on the underlying Canadian population; however, when weighting was explored, minor differences in proportions were observed. While the survey was investigator-developed and had not gone through psychometric testing, it was reviewed extensively, and beta-tested by patient partners and medical cannabis experts. The possibility of the survey being completed inauthentically by a computer program (i.e., bot) must be acknowledged. Several preventive strategies were employed including requiring human authentication, the use of enhanced fraud detection software embedded in Qualtrics©, and the detection of multiple responses obtained from the same device.

## Conclusions

With the advent of cannabis legalization in Canada, there has been increasing interest in the impact on public health outcomes, specifically medical cannabis access and use. Our study is among the largest conducted in Canada since legalization that explores the demographic and health- and medical cannabis-related factors associated with holding authorization, as well as differences in medical cannabis use and experiences between those with authorization and those without. The findings shed light on possible inequities experienced by Canadians in accessing medical cannabis authorization following the legalization of recreational cannabis, as well as the potentially positive impacts of obtaining medical cannabis authorization from a healthcare professional. Further research is needed to continue to explore the implications of recreational cannabis legalization on how medical cannabis is used, accessed, and experienced.

### Supplementary Information

**Supplementary Material 1.**

## Data Availability

Data for this study is not available due to privacy concerns and sensitivity around the topic. Statistical coding can be made available upon request.

## Abbreviations

ACMPR: Access to Cannabis for Medical Purposes Regulations
CBD: Cannabidiol
CCS: Canadian Cannabis Survey
CI: Confidence intervals
OR: Odds ratio
SD: Standard deviation
THC: Tetrahydrocannabinol

## References

[1] Whiting PF, Wolff RF, Deshpande S (2015). Cannabinoids for medical use: a systematic review and meta-analysis. JAMA..

[2] National Academies of Sciences, Engineering, and Medicine; Health and Medicine Division; Board on Population Health and Public Health Practice; Committee on the Health Effects of Marijuana: An Evidence Review and Research Agenda. The Health Effects of Cannabis and Cannabinoids: The Current State of Evidence and Recommendations for Research. Published online January 12, 2017. Accessed 24 Mar 2023. https://www.ncbi.nlm.nih.gov/books/NBK425767/.

[3] Government of Canada. Information for health care professionals: cannabis (marihuana, marijuana) and the cannabinoids. 2018. Accessed March 21, 2023. https://www.canada.ca/en/health-canada/services/drugs-medication/cannabis/information-medical-practitioners/information-health-care-professionals-cannabis-cannabinoids.html.

[4] Government of Canada. Cannabis Act. 2018. Accessed July 3, 2023. https://laws-lois.justice.gc.ca/eng/acts/c-24.5/.

[5] Government of Canada. Canadian Cannabis Survey 2022. 2022. Accessed March 21, 2023. https://www.canada.ca/en/health-canada/services/drugs-medication/cannabis/research-data/canadian-cannabis-survey-2022-summary.html#s5.

[6] Government of Canada. Data on cannabis for medical purposes. Accessed January 30, 2024. https://www.canada.ca/en/health-canada/services/drugs-medication/cannabis/research-data/medical-purpose.html.

[7] Walsh Z, Callaway R, Belle-Isle L (2013). Cannabis for therapeutic purposes: patient characteristics, access, and reasons for use. Int J Drug Policy.

[8] Kendzor DE, Ehlke SJ, Kharazi Boozary L, Smith MA, Cohn AM (2022). Characteristics of adults with a medical cannabis license, reasons for use, and perceptions of benefit following medical cannabis legalization in Oklahoma. Prev Med Rep.

[9] Lintzeris N, Mills L, Abelev SV, Suraev A, Arnold JC, McGregor IS (2022). Medical cannabis use in Australia: consumer experiences from the online cannabis as medicine survey 2020 (CAMS-20). Harm Reduct J.

[10] Sznitman SR (2017). Do recreational cannabis users, unlicensed and licensed medical cannabis users form distinct groups?. Int J Drug Policy.

[11] Minhas M, Lunn SE (2022). Letter to the editor: the need for equitable health care among medical cannabis patients in Canada. Cannabis Cannabinoid Res.

[12] Government of Canada. Cannabis for medical purposes. Published May 30, 2023. Accessed July 3, 2023. https://www.veterans.gc.ca/eng/about-vac/research/research-directorate/publications/reports/cmp.

[13] Health Canada. *Access to cannabis for medical purposes in Canada: gathering information on views and practices of patients and health care practitioners*; 2022. Accessed March 21, 2023. https://epe.lac-bac.gc.ca/100/200/301/pwgsc-tpsgc/por-ef/health/2022/093-21-e/POR093-21-Report.pdf.

[14] Asselin A, Lamarre OB, Chamberland R, McNeil SJ, Demers E, Zongo A (2022). A description of self-medication with cannabis among adults with legal access to cannabis in Quebec, Canada. J Cannabis Res.

[15] Kruger DJ, Kruger JS, Collins RL (2020). Cannabis enthusiasts’ knowledge of medical treatment effectiveness and increased risks from cannabis use. Am J Health Promotion.

[16] Cameron J, Dhalla R, Lougheed T, Blanc A, Vaillancourt R (2023). An examination of cannabis-related information typically asked by consumers at retail cannabis locations: a Canadian survey of budtenders and managers. Canadian Pharmacists Journal / Revue des Pharmaciens du Canada.

[17] Capler NR, Balneaves LG, Buxton JA, Kerr T (2023). Reasonable access: important characteristics and perceived quality of legal and illegal sources of cannabis for medical purposes in Canada. J Cannabis Res.

[18] Kruger DJ, Gerlach J, Kruger JS, Mokbel MA, Clauw DJ, Boehnke KF. Physicians’ attitudes and practices regarding cannabis and recommending medical cannabis use. Cannabis Cannabinoid Res. Published online April 25, 2023. 10.1089/can.2022.0324. 10.1089/can.2022.0324 PMC11538087 37098170

[19] Shulman H, Sewpersaud V, Thirlwell C (2022). Evolving global perspectives of pharmacists: dispensing medical cannabis. Cannabis Cannabinoid Res..

[20] Canadian Pharmacy Association (2018). Issues spotlight: medical cannabis. Canadian Pharmacists Journal / Revue des Pharmaciens du Canada.

[21] Dattani S, Mohr H (2019). Pharmacists’ role in cannabis dispensing and counselling. Canadian Pharmacists Journal / Revue des Pharmaciens du Canada.

