# Supplementary material for: Canadians’ use of cannabis for therapeutic purposes since legalization of recreational cannabis: a cross-sectional analysis by medical authorization status
Source: BMC Med. 2024 Apr 8;22:150. doi: 10.1186/s12916-024-03370-7 (PMC11003000; doi:10.1186/s12916-024-03370-7)
Supplement: Supplementary file 1 — Supplementary Material 1. [file 12916_2024_3370_MOESM1_ESM.docx]

Canadians’ use of cannabis for therapeutic purposes since legalization of recreational cannabis: a cross-sectional analysis by medical authorization status

**Supplementary Methods**

*Demographic and health-related variables:*

- Age – Participants were asked to input their age in an open text box. From this, they were categorised into six groups: <30 years, 30-39.9 years, 40-49.9 years, 50-59.9 years, 60-69.9 years, and ≥70 years of age.
- Canadian province or territory – Participants were asked “What province or territory do you currently live in?” Based on their responses, they were categorized into the respective province or territory where they lived.
- Ethnicity – Participants were asked “Which of the following ethnic/racial groups do you identify with?” and could select multiple options on a list. From these responses, participants were categorized into six categories: White, Black, Asian, Indigenous, Latin American, and Mixed (if more than one ethnicity selected). If participants did not answer, they were categorized as “Unknown” ethnicity.
- City/Town – Participants were asked “The area you live in is…”, with the options being: large city (>500,000 people), medium city (200,001-499,999 people), small city (10,000-200,000 people), or small town or rural/remote area (<10,000 people).
- Gender – Participants were asked what their gender was, in which they could select: Man, woman, non-binary, or not listed. From this, participants were classified into their respective gender or included into an unknown category.
- Education – Participants were asked what their highest level of education was, with answers classified into five categories: No diploma or degree; High school; College, trade certificate, or diploma; Undergraduate degree; and Graduate degree (i.e., master’s or PhD).
- Household income – Participants were asked what their annual household income (before taxes) was and could select: <$35,000, $35,000 to $50,000, $50,001 to $75,000, $75,001 to $100,000, $100,001 to $150,000, >$150,000. Based on responses, participants were categorized into the following groups:  <$50,000 per year, $50,001-75,000 per year, $75,001-100,000 per year, >$100,001 per year, or an unknown/missing category.
- Canadian Armed Forces – Participants were asked “Are you currently, or have you ever been, a member of the Canadian Armed Forces, including the reserve forces?” They were categorized based on their responses of “Yes”, “No” or “Unknown” if they did not answer the question or selected “Prefer not to answer”.

*Reasons for taking medical cannabis*

Participants were asked if the reasons they use medical cannabis and could select multiple options from the following list:

- In addition to other medication to treat my symptoms or health conditions
- To reduce my use of other medication(s) used to treat my symptoms or health conditions
- To reduce side effects of other medications or treatments
- As other medication(s) do not work well for me
- As part of a general health and wellness routine
- At times for recreational purposes (e.g. for enjoyment or social reasons)
- It works well managing my health condition(s)/ symptom(s)
- It enhances the effect(s) of other medications or treatments I take
- It works better than other medications or treatments I have take
- It’s a natural treatment
- It gives me control over my health
- It’s less expensive than my other medications
- It has fewer side effects than other medication I take or took
- I can purchase it over the counter at a recreational store

*Side effects from taking medical cannabis*

All participants were asked if they experienced any unwanted side effect from taking medical cannabis and they could select multiple options from the following list:

- Anxiety
- Confusion
- Cough
- Dependency or addiction to cannabis
- Dry mouth
- Feeling faint
- Feeling intoxicated (high)
- Feeling paranoid
- Feeling tired
- Rapid heart rate
- Trouble remembering things
- Nausea
- Unable to concentrate
- Vomiting

If participants did not experience any side effects they had the option of selecting “I have not experience any side effects” at the bottom of the list.

##### **Table S1.** Health conditions and symptoms managed by current medical cannabis consumption

| **Health Condition or Symptom** | **Currently take medical cannabis** | |
| --- | --- | --- |
|  | **With authorization** | **Without authorization** |
| ADHD | 335 (11.6%) | 489 (19.9%) |
| Agitation | 377 (13.0%) | 478 (19.4%) |
| Anxiety | 1666 (57.6%) | 1738 (70.7%) |
| Appetite | 590 (20.4%) | 770 (31.3%) |
| Autism spectrum disorder | 85 (2.9%) | 92 (3.7%) |
| Bipolar disorder | 116 (4.0%) | 134 (5.4%) |
| Cancer | 99 (3.4%) | 81 (3.3%) |
| Cancer related pain | 79 (2.7%) | 69 (2.8%) |
| Colitis | 55 (1.9%) | 60 (2.4%) |
| Crohn's | 82 (2.8%) | 58 (2.4%) |
| Concentration | 351 (12.1%) | 391 (15.9%) |
| Diabetes | 126 (4.4%) | 106 (4.3%) |
| Depression | 1204 (41.6%) | 1406 (57.2%) |
| Epilepsy | 51 (1.8%) | 32 (1.3%) |
| Irritable bowel syndrome | 497 (17.2%) | 418 (17.0%) |
| Pain - Acute | 509 (17.6%) | 516 (21.0%) |
| Pain – Chronic | 2115 (73.1%) | 1470 (59.8%) |
| Migraine | 862 (29.8%) | 801 (32.6%) |
| Muscle spasms | 1020 (35.3%) | 771 (31.3%) |
| Nausea and vomiting | 612 (21.2%) | 750 (30.5%) |
| Obesity | 103 (3.6%) | 106 (4.3%) |
| PCOS | 68 (2.4%) | 90 (3.7%) |
| PTSD | 644 (22.3%) | 618 (25.1%) |
| Seizures | 71 (2.5%) | 39 (1.6%) |
| Sleep issues | 1771 (61.2%) | 1536 (62.4%) |
| Stress | 1316 (45.5%) | 1305 (53.0%) |
| Traumatic brain injury | 138 (4.8%) | 66 (2.7%) |
| Abbreviations: ADHD, attention-deficit/hyperactivity disorder; PCOS, polycystic ovary syndrome; PTSD, post-traumatic stress disorder | | |
